# A reach-out system for video microscopy analysis of ciliary motions aiding PCD diagnosis

**DOI:** 10.1186/s13104-015-0999-x

**Published:** 2015-03-08

**Authors:** Israel Amirav, Huda Mussaffi, Yehudah Roth, Miriam Schmidts, Heymut Omran, Claudius Werner, for the Israeli PCD Consortium Investigators

**Affiliations:** Ziv Medical Center, Bar Ilan University, Safed, Israel; Schneider Children’s Medical Center, Petach-Tikva, Israel; Edith Wolfson Medical Center, Holon, Israel; Genetics and Genomic Medicine, Birth Defect Research Center, Institute of Child Health, University College London (UCL), London, UK; Human Genetics Department, Radboudumc, Nijmegen, The Netherlands; Department of Pediatrics Muenster, University Hospital Muenster, Muenster, Germany; Stollery Children’s Hospital, Faculty of Medicine, University of Alberta, Edmonton, AB Canada

**Keywords:** Cilia, Video, Microscopy, Portability

## Abstract

**Backgrounds:**

High-speed Video-Microscopy Analysis (HVMA) is now being used to aid diagnosis of Primary Ciliary Dyskinesia (PCD). Only a few centers however, are equipped with the available resources and equipment to perform these tests. We describe our experience in HVMA reaching-out to many more peripheral and relatively remote areas.

A portable computer with HVMA software, video camera and a microscope were used. Fourteen disperse pediatric centers were reached and a total of 203 subjects were tested within a relatively short time (Clinical Trial Registration: NCT 01070914 (registered February 6, 2010).

**Results:**

With an average time of 20 minutes per patient, the system enabled us to test approximately 10–15 subjects per day. A valid HVMA result was made in 148 subjects and helped in the diagnosis of PCD in many of the patients who were subsequently confirmed to have PCD by electron microscopy and/or immunofluoresence and/or genetics and/or nasal Nitric Oxide testing. The sensitivity of abnormal HVMA to accurately predict PCD was 90.2%.

**Discussion and conclusion:**

This is the first report of an out-reach system to record HVMA for improved diagnosis of PCD in remote regions that are not within reach of PCD centers and experts. It provides immediate preliminary results and instantaneous feedback to the physician, patient and his/her family members in these areas. Future studies to compare this system to conventional desk top systems are warranted.

**Trial registration:**

NCT 01070914 (registered February 6, 2010).

## Introduction

Primary ciliary dyskinesia (PCD) is an inherited disorder characterized by recurrent infections of the upper and lower respiratory tract, reduced fertility in males and situs inversus in about 50% of affected individuals [1,2]. Respiratory ciliary motility dysfunction is a major feature of PCD and has been traditionally evaluated by simple light microscopy of ciliated epithelium cells. Although this approach may identify markedly diminished or absent beat frequency, ciliary beating may appear normal in some cases of PCD [1,3]. Moreover, it has become apparent that assessment of both ciliary beat pattern and frequency are essential as cilia may beat in a dyskinetic fashion while maintaining their normal beat frequency [4,5]. Therefore, the use of conventional light microscopic assessment of ciliary motility is no longer recommended as a screening technique. With recent advances in High-speed imaging, High-speed Video-Microscopy analysis (HVMA) evaluating both ciliary beat pattern and frequency is now used as part of routine diagnostic testing of PCD [1,4,5]. With this method a camera is attached to a microscope and the brushing specimen are placed under the microscope, after which images are recorded and analyzed. Only a handful of laboratories are equipped with the available resources and specialized equipment to perform these tests. Highly centralized centers equipped with HVMA technologies have been developed [6] where patients are referred for diagnostic purposes.

As an alternative to the patients travelling to the center it has been suggested that samples taken at the patient’s local point of care be transported to the diagnostic center. This also has many drawbacks such as sample degradation due to transport delays as well as problems with standardization of procedures.

The aim of this paper is to describe our experience implementing a reach out system in order to enhance and complement diagnostic work-up for PCD in remote areas usually lacking access to sophisticated diagnostic settings.

## Methods

A study to characterize all known and suspected PCD patients in Israel (NCT 01070914) is currently being undertaken in Israeli pediatric pulmonology centers using multiple diagnostic PCD tests. The study is in compliance with the Helsinki Declaration and ethical approval was obtained from the local research committee at each centre that collected patients’ data (Ziv Medical Center, Schneider Children’s Medical Center, Edith Wolfson Medical Center, Soroka Medical Center, Hadassah-Hebrew University Medical Centers, Rambam Medical Center, Western Galilee Hospital, Saint Vincent De-Paul Hospital, Assaf Harofeh Medical Center, Shaare Zedek Medical Center, Edmond & Lili Safra Children’s Hospital, Sheba Medical Center, Carmel Medical Center, Dana Children’s Hospital) as well as from the Israeli Ministry of Health. Informed consent was obtained from each subject or his/her guardian. Inclusion criteria were 1. Diagnosis of PCD (mostly clinical diagnosis and sometimes other partial ancillary test results, mainly electron microscopy) or 2. Suspected PCD based on unexplained Situs Inverses, chronic pulmonary or ENT symptoms and/or signs, fertility disorders or family members with suggestive clinical history of PCD.

Following recruitment each subject completed an in depth clinical history, before undergoing nasal brushing [for Electron Microscopy (EM), Immuno-fluorescence (IF) and video-microscopy], blood sampling (for genetic analysis) and measurement of nasal Nitric Oxide (NO). Thus, each subject underwent all of these 5 tests including an HVMA evaluation of his/her cilia. The nasal brushing material was first used for HVMA and the remaining was used for IF staining and EM. NO measurements were obtained according to established American Thoracic Society guidelines. A single manufacture unit (CLD 88 SP, Eco Physics Duernten, Switzerland) was used in all centers. Most of the NO tests were done by a single team of investigators (MP, DB) with the same unit. Some of the NO tests were done by the experienced technicians at local centers using identical standardized operating procedures (Single breath with breath hold). The average of three measurements was used for statistical analysis. In young children (usually less than 4 years) the tidal breath method was employed. The analyzers were calibrated according to the manufacturer’s specifications.

The procedure for HVMA started by obtaining respiratory epithelial cells from the patient by nasal brushing using a conventional cervix cytology brush [Celletta™ brush cell collector with protective tip (product number 9100060; Engelbrecht Medizin-und Labortechnik GmbH, Tiefenbachweg 13; 34295 Edermünde, Germany)]. The first author has been trained in nasal brushing by experienced investigators (HO, CW, MS) before the present study. To minimize pain, the brush was rinsed with an isotonic saline solution before being inserted in the inferior nasal meatus, using rotatory and linear movements. The brush was immediately removed and transferred into a tube containing 2–3 mL of cell culture medium (RPMI Medium 1640, Endotoxin tested, Cell culture tested, without L-Glutamine, without Sodium Bicarbonate. Biological Industries LTD., Beit Haemek, Israel). The tube was then shaken vigorously so that cells became detached from the brush. The brush was withdrawn from the tube and used to smear slides (for later IF analysis) while the last portion of the material on the brush was stored in a glutaraldehyde filled tube for later EM analysis.

**Figure 1:**
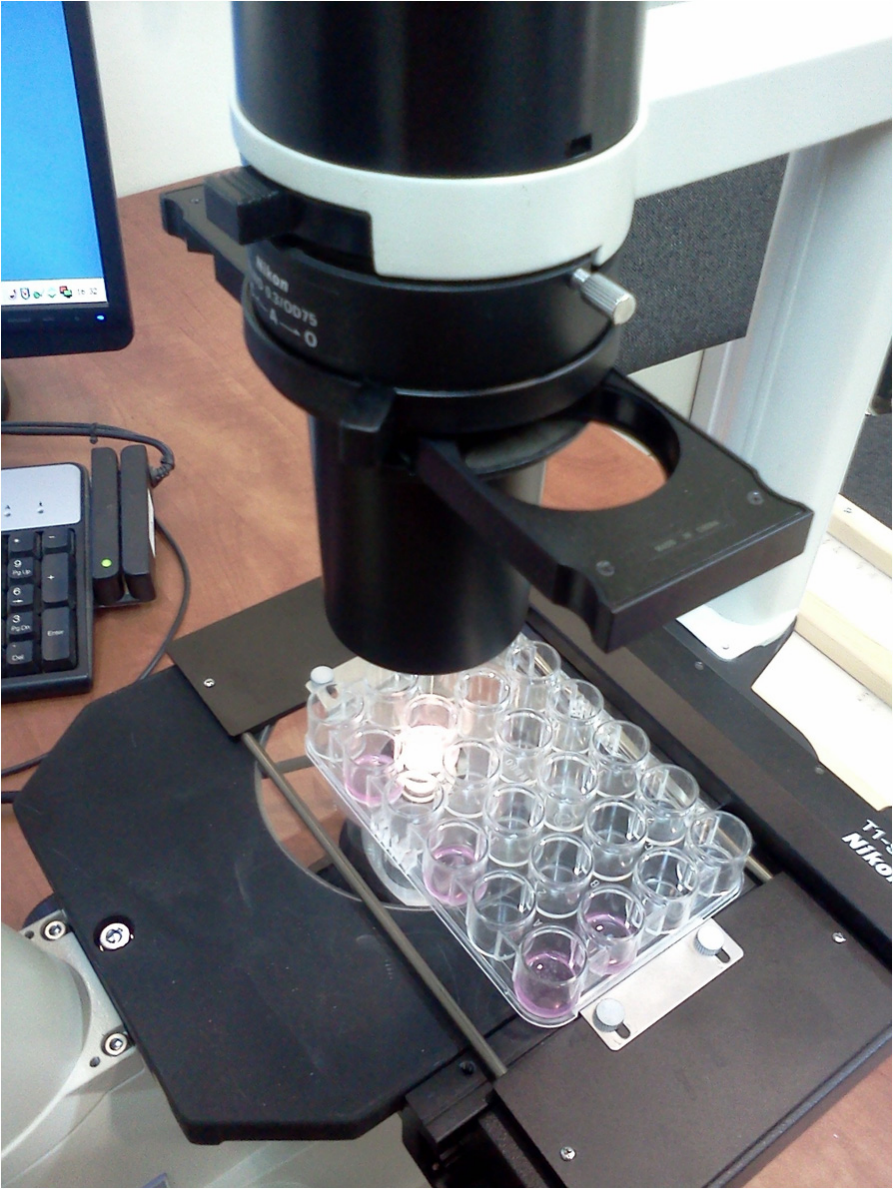
**Use of a 24 well plate for microscopy.**

A plastic Pasteur pipette was then used to draw approximately 50 microliter fluid sample from the RPMI tube and put it in one of the wells of a multi-well cassette under the microscope. Traditional HVMA uses single slides per sample and each fluid sample is put on a new slide for the HVMA. In this case we used a Linbro multiwell plate (Figure 1, Hampton Research Corp., CA, USA). This is a 24 well plate where the wells were identified by lettered rows A through D and numbered columns 1 through 6. It allowed multiple fluid samples to be placed in the wells with no need to remove the plate from the microscope holder between patients. The use of the multi-well plate instead of individual glass slides minimized microscope movements (during slides changes) and the need for focus adjustments.

**Figure 2:**
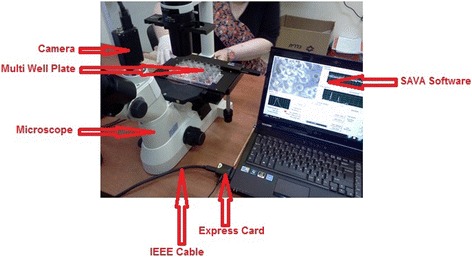
**Components of the HVMA system.**

All sample images were visualized with a relatively small Eclipse TS-100 inverted phase contrast microscope (Nikon Corp. Tokyo, Japan) using a 40X objective lens (numerical apparatus = 0.55) providing a total magnification of 400 X. Eight to ten video images of each sample under the microscope were selected for recording based upon the visibility of vital ciliated cells, good resolution and preferably, the presence of cell strips. Images were recorded using a scA640-120 fm digital video camera (Basler AG, Ahrensburg, Germany) attached to the microscope. The camera was connected to a laptop computer (LG model #R58) with an IEEE cable. The computer was equipped with a high power video card and an Express card IEEE1394B interface (Figure 2). The laptop computer was easily transportable and the whole system was packed on a heavy duty trolley (Pelican 1630, Pelican Products, Inc., CA, USA) for transport.

All image recordings and processing were performed with the Sisson-Ammons Video Analysis (SAVA) system [7], which was highly customized to perform ciliary analysis for both ciliary beating frequency (CBF) and patterns. Images were captured initially into RAM in real-time at 120 fps and immediately compressed and stored to disk. At a sampling rate of 120 fps, approximately 3 s of video were recorded for each image. Each digital image frame consisted of 640 columns X 480 rows of pixels.

Images were recorded at room temperature within 10 minutes after brushing. Once recorded, the video clips for each subject (and sample) were played back in normal time and slow motion (up to 1/8th speed) for quality assessment and analysis of beat frequency and pattern. If the quality was deemed inadequate, we used the pipetor to draw a second sample from the tube into another well. If this was also insufficient, a second nasal brushing was performed on the subject and the process was repeated.

Once this process was deemed satisfactory, the subject had completed his test and the system was immediately ready for the next subject. An initial *preliminary* assessment of the video clips was done on-site and a general subjective impression (general 3 categories of normal or likely normal; abnormal or possibly abnormal; and undetermined) was immediately shared with the treating physicians (and also with the patient and his/her family) who were usually present at the site but may not always attend similar tests done in centralized institutions. With a few simple clicks in the SAVA software, the next subject’s details appeared on the screen and the system was ready for the next subject’s sample.

A much more thorough and comprehensive detailed analysis of the video clips was performed post hoc at the central laboratory where each video clip was played back and analyzed separately and a final result of findings was prepared. This analysis and interpretation was done by 3 experts in HVMA who were not involved in the data collection (2 from Germany, 1 from the UK) and were blind to the clinical data, NO EM, IF or genetic results of the subject. In cases of ambiguity the files were further re-analyzed and final report was reached by consensus. Analysis of each video clip included quality of image (images with no epithelia cells and/or clips with only single cells were excluded from further analysis), and beating pattern. Reviewing all 8–10 images for each subject and taking into account all of these criteria, the HVMA results of each subject were then categorized into the following specific categories [8]: Normal or Probably Normal beating (regular forward and recovery strokes); Immotile cilia; Residual movement (almost immotile cilia with minimal movements); Stiff beating (due to reduced bending capacity/amplitude); Abnormal circular beating; Hyperkinetic cilia; No video available; Cannot be assessed. The last category (cannot be assessed) included subjects with poor quality clips for different causes such as those with single (not within an epithelial strip) immotile ciliated cells, poor resolution due to cells entrapped in mucus or subjective inability to distinguish between cilia of dying or damaged cells (secondary dyskinesia) to dyskinesia or a-kinetic cilia in a vital cell (primary dyskinesia). In these cases, nasal brushing and HVMA should be repeated three to six months later.

The diagnosis of PCD was confirmed following the completion of the study and based on assessment of all available tests for each individual. In addition to pre-determined classic clinical characteristic as an inclusion criteria, confirming the diagnosis of PCD in this study required at least 2 abnormal tests out of the 5 tests performed (EM, IF, Genetics, NO and HVMA). The distribution of various HVMA findings in the PCD diagnosed patients were then summarized post-hoc.

The sensitivity of HVMA as an index test to accurately diagnose PCD was calculated as the proportion of abnormal HVMA in the total number of confirmed PCD. For the purpose of sensitivity calculation, PCD was confirmed by the presence of at least two abnormal references tests out of four (EM, IF, Genetics, NO; without the index test-HVMA)]. As there is no gold standard for PCD [9] it is very challenging to *exclude* PCD and to calculate specificity and/or negative predictive value of diagnostic tests. For example, even when PCD is defined by abnormal genetics, it is impossible to define “non PCD” (true negative, false positive) cases, as a subject who is negative for known PCD mutations can still harbor a new unrecognized PCD causing mutation. Therefore, we have focused on calculating the sensitivity of HVMA, namely the probability of an abnormal HVMA in subjects with PCD.

## Findings and results

Using this mobile system we have so far performed HVMA tests in 203 subjects in 14 different pediatric pulmonology centers throughout the country. With an approximate 20 minutes testing time per patient, from brushing to complete analysis of the ciliary beating pattern, we were able test 10–15 subjects per day. Apart from individual transport time to each center location, it took another hour to reassemble and disassemble the system in each location.

**Figure 3:**
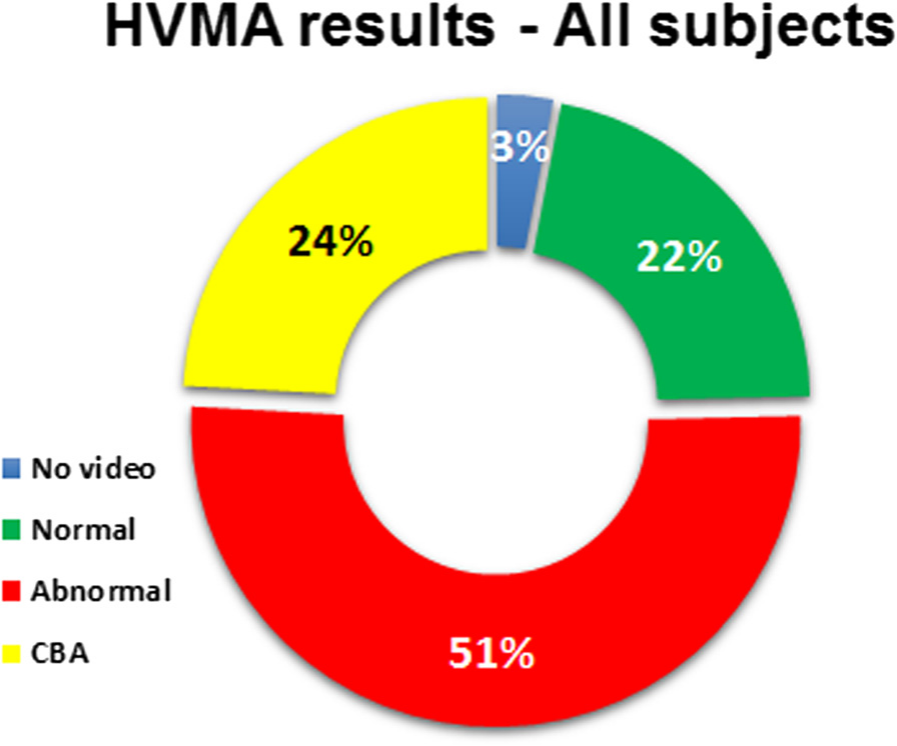
**HVMA on-site results (percentage of each category) in all subjects (n = 203) (CBA = cannot be assessed).**

**Figure 4:**
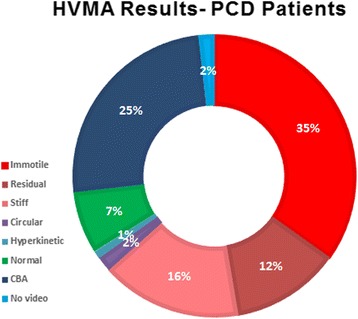
**HVMA post-hoc results (percentage of each category) in PCD patients (n = 112).**

Failure to obtain epithelial cells was infrequently observed on initial testing and gradually disappeared later on. Although not formally assessed, we have noticed decreased variability and increased expertise in both brushing and video analysis over time. Altogether, approximately 1500 video clips were analyzed. Six subjects had no adequate samples and/or no video recording. In another 49 subjects, a HVMA result was determined to be “cannot be assessed”. An HVMA result was made in 148 out of 203 (73%) subjects. The results of HVMA are depicted in Table 1, Figure 3 (on site results in all subjects) and Figure 4 (post hoc results in PCD patients). Although the HVMA categories were different between the initial on-site assessment (3 general categories) and the post-hoc assessment (7 specific categories), there was a high degree of agreement between the two, with only 6 subjects whose final post-hoc results were not within the initial on-site general categories.

**Table 1:** **Results of post –hoc and on-site HVMA in all study subjects (n = 203) and in PCD patients (n = 112) (CBA = cannot be assessed)**

	**Post Hoc**		**On-Site**	
**Category**	**All**	**PCD**	**All**	**PCD**
**Immotile**	45	39	47	41
**Residual**	15	14	15	14
**Stiff**	36	18	37	18
**Circular**	6	2	6	2
**Hyperkinetic**	2	1	2	1
**Normal**	44	8	44	8
**CBA**	49	28	46	26
**No video**	6	2	6	2

PCD was confirmed in 112 subjects. Detailed results of HVMA of these 112 PCD patients (which were diagnosed in our study according to the previous definition), are depicted in Table 1 and Figure 4.

The sensitivity of on-site abnormal HVMA (index test) to accurately predict PCD (defined as at least 2 abnormal reference tests out of 4) was 90.5%. It was not different from the post-hoc HVMA sensitivity (90.2%).

Although no diagnosis of PCD was made on-site, inviting the parents, primary physician and/or the patient to view the video on-site within minutes after the brushing was highly appreciated and rewarding for many of them.

## Discussion

This is the first report of an out-reach system to record HVMA of cilia for improving diagnosis of PCD. The portable system was used successfully in the evaluation of patients in regions that are not within easy reach of PCD centers and experts. Currently in Israel, there is only one center equipped with a stationary HVMA and many patients, as well as physicians, are reluctant to bear the travel expense and long time constraints involved. According to a recent study in 290 centers treating PCD throughout Europe [10], only 27% reported being able to perform HVMA varying from 52% in the British Isles to only 7% in eastern Europe. As diagnosis of PCD remains difficult and confined to specialized centers, [11] the present method allows for better access to remote places and allows patients to be tested close to or even in their hometown without the need for long distance travel.

The results of the present study demonstrate that HVMA using a reach-out system can be used as a complementary test to aid in the diagnosis of PCD. Sensitivity of the reach-out HVMA system to predict PCD in this study was 90%. It was no different from the sensitivity calculated using off-site HVMA. It is interesting to note the sensitivity measured in the present study was similar to the sensitivity of HVMA (defined as “dyskinesia score equal or exceeding 2”) to predict PCD reported by Stannard et al. [4].

It must be acknowledged that we have used only a single brushing which may have over-estimated the true abnormal cases of PCD due to potential inclusion of secondary motion abnormalities. Thus, the current calculated sensitivity of the test may be under-estimated. For clinical purposes however, the inclusion of other tests in these cases reduced this potential drawback.

Portability and access to diagnostic tests is of great importance particularly with rare diseases. It has been known that PCD is underdiagnosed [2,12]. The effects of under-diagnosis may have a stronger impact in remote areas where inhabitants have little chance of being diagnosed and treated properly without leaving their jobs and travelling long distances, sometimes taking days to reach the closest PCD referral center. Wrong diagnosis in patients with bronchiectasis can be harmful, because other disorders (e.g. Common variable immunodeficiency syndromes) are treated differently than PCD, and timely diagnosis and early treatment may produce a decrease in morbidity [13].

PCD care requires a reasonable compromise between centralized treatment by highly specialized expert teams at large time intervals and frequent visits at small local centers with limited expertise. Our portable HVMA offers complementary state-of-the-art PCD diagnostics to patients and, at the same time, provides specialized knowledge to physicians at smaller centers. This is ideal for regions with few, scattered experienced centers and reluctance of patients to travel long distances to undergo diagnostic testing. However, in societies, where travelling is not an important obstacle, a portable HVMA system will not be of additional value.

Due to the identical analysis methodology used in a stationary system, the current portable HVMA system, although smaller and much less expensive than fixed systems, should not lack in relative quality or functionality. The ease of use is also a big advantage. Thus, the technological aspects of the system may, in the future, allow rural sites to achieve tertiary site image quality with basic training. From our experience it can be estimated that 50 nasal brushings should be practiced to acquire good hands on expertise. A dedicated physician, technician or nurse with appropriate training and could be the core of future similar reach out teams. Gaining of expertise by the team in performing the tests will further strengthen the system.

Of course there is a value in centralized care and diagnosis, yet our current approach also takes the patients’ needs and satisfaction into account. Although we have no data to compare, it is a reasonable assumption that patients’ rate of compliance with testing may be higher compared to their compliance when using a long distance center.

The use of a single plastic plate containing multiple wells allows us to save a lot of time. There is no need to open the microscope slide holders at all, the plastic plate remains in place during the whole visit at the clinic or hospital, and it is also more economical.

The video acquiring system could be operated by one or two technicians travelling from site to site. Data could be sent on-line back to a doctor or a central laboratory if further analysis is necessary.

In this specific study, in addition to HVMA on-site testing, the NO measurements were also performed on-site using a single manufacture large unit (CLD88). In the future, with the advance in portable NO analyzers, reach-out systems may also include portable NO analyzers, further strengthening the diagnostic yield of a reach-out system.

Some limitations need to be acknowledged: when the number of subjects per center is small, travelling to a remote area may be economically inefficient. However, a formal economic comparison with office based testing was beyond the scope of our study. As PCD is a rare disease, it is likely that the number of subjects requiring PCD diagnostic testing will decrease after most previously undiagnosed individuals have been identified. This is a welcome effect accompanying any introduction of standardized care in rare diseases. Another technical limitation of our study is that temperature is not controlled in the approach described. That means that, in general, the temperature is around 24°C due to the heat generated by the microscope. Some researchers believe it is important to perform HVMA at 37°C [14]. We do not consider it a major drawback as, in our experience, as well as that of others [15], ciliary beat *patterns* do not significantly vary at different temperatures. Furthermore, lower temperature reduces beat *frequency* which in turn allows better visualization and capturing of very fast beat variants. [8] The “lower” (x400) magnification in our portable system may reduce resolution compared to other systems. However, as shown recently by our group, higher magnification markedly reduced depth of focus in the Z plane and limits ciliary motion interpretation (see video s20 in Ref. [8]). The magnification used in the present portable system is identical to the stationary one used routinely in our Germany center [8]. Standardization of HVMA is crucial in both stationary and reach-out system and this will require more discussion and consensus [9].

## Conclusions

We have described our successful experience with a new reach-out HVMA system for PCD diagnosis. The system may improve quality of care by enabling large scale testing to be efficiently done in any remote or non-remote center, enabling HVMA to be accessible to a greater number of patients suspected of suffering from PCD. Future studies to compare this system to conventional desk top systems are warranted.

## Abbreviations

HVMA: High speed video microscopy analysis
PCD: Primary ciliary dyskinesia
CBF: Ciliary beat frequency
